# Three-Dimensional Dual-Energy Computed Tomography for Enhancing Stone/Stent Contrasting and Stone Visualization in Urolithiasis

**DOI:** 10.1155/2013/646087

**Published:** 2013-07-17

**Authors:** El-Sayed H. Ibrahim, William E. Haley, Maria A. Jepperson, David D. Thiel, Michael J. Wehle, Joseph G. Cernigliaro

**Affiliations:** ^1^Department of Nephrology and Hypertension, Mayo Clinic, 4500 San Pablo Road, Jacksonville, FL 32224, USA; ^2^Department of Radiology, Mayo Clinic, 4500 San Pablo Road, Jacksonville, FL 32224, USA; ^3^Department of Urology, Mayo Clinic, 4500 San Pablo Road, Jacksonville, FL 32224, USA

## Abstract

The use of dual-energy computed tomography (DECT) for evaluating urinary calculi has been appreciated due to the modality's capability of differentiating between uric acid (UA) and non-UA stones, which are color coded based on a postprocessing algorithm. No other imaging modality or laboratory test is able to identify the stone composition without first attaining the stone material. Knowledge of the stone composition is clinically significant since UA calculi may be treated medically whereas non-UA calculi may require surgical removal. Regardless of the stone type, ureteral stents are often placed to prevent or treat obstruction. Recent work has demonstrated that commonly used stents are also colored based on their dual energy characteristics and may thereby either improve or obscure the identification of adjacent calculi. Herein, we report the case of a 65-year-old man who underwent percutaneous nephrolithotomy of a large staghorn stone with subsequent significant residual stone fragments noted on a follow-up scan. By using three-dimensional DECT and taking advantage of color contrasting, the stone composition, burden, shape, and boundary were clearly depicted apart from the adjacent stent, resulting in successful medical treatment and obviating the need for further surgical intervention.

## 1. Introduction

Dual-energy computed tomography (DECT) is a recently introduced technique for imaging urinary calculi that acquires image data simultaneously from two X-ray tubes with different tube potentials, yielding different attenuation characteristics of the same tissue [1, 2]. The ratio of these two attenuations uniquely differentiates between uric acid (UA) and non-UA stones, which are color coded on the reconstructed DECT image (UA and non-UA stones appear red and blue, resp., on Siemens scanners, as shown in this report). DECT has demonstrated the ability to instantly distinguish UA from non-UA stones larger than 3 mm with near 100% specificity [3–5]. It is notable that the DECT technology thereby introduced the capability of identifying the stone composition without first having to attain the stone material, the clinical significance being that this informs about the treatment strategy: while UA stones may be treated medically, non-UA stones may require surgical intervention [6, 7]. 

Ureteral stents, which are often placed to prevent or treat obstruction, are also characterized by the dual-energy (DE) algorithm; those with change in attenuation similar to UA stones are colored in red while those with change in attenuation similar to non-UA stones are colored in blue. A list of the DE characteristics of commonly used ureteral stents has previously been reported [8]. Imaging of stents with DECT may either improve or obscure the identification of adjacent calculi, depending upon the colors assigned to them according to their DE characteristics. 

## 2. Case Report

A 65-year-old male with history of gout underwent a percutaneous nephrolithotomy to remove a large staghorn kidney stone. A 3-dimensional DECT scan was utilized to evaluate procedural results. The images were acquired on a Somatom Definition Flash CT scanner (Siemens Healthcare, Forchheim, Germany). The tube potentials (kVp) were set to 80 kV and 140 kV, and the scan was conducted using a dedicated renal stone imaging protocol. Continuous noncontrast images were obtained from the level of the diaphragms to the pubic symphysis. The images were constructed in the axial and coronal planes with 1 mm slice thickness and 0.8 mm slice interval using D30f convolution kernel. Image postprocessing was conducted on a multimodality workstation provided by the manufacturer using the Syngo software (version VE36A). The postprocessing algorithm used a 3-material (UA, calcium, and urine) decomposition algorithm to assign color (red or blue) based on the ratio of X-ray attenuations from the two tube potentials.

**Figure 1:**
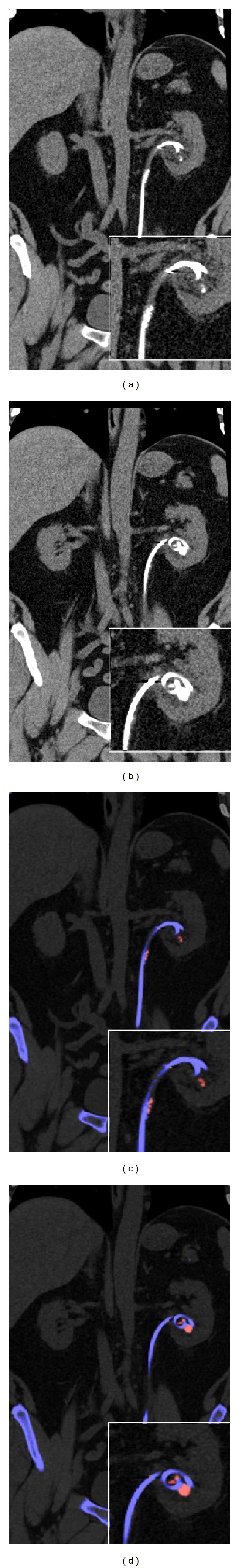
Two-dimensional coronal conventional computed tomography (CT) ((a), (b)) and dual-energy computed tomography (DECT) ((c), (d)) images showing kidney stones (magnified insets) and nephrostomy stent. The stones and stent have the same contrast in conventional CT, while, in DECT, the stones and stent appear red and blue, respectively.

**Figure 2:**
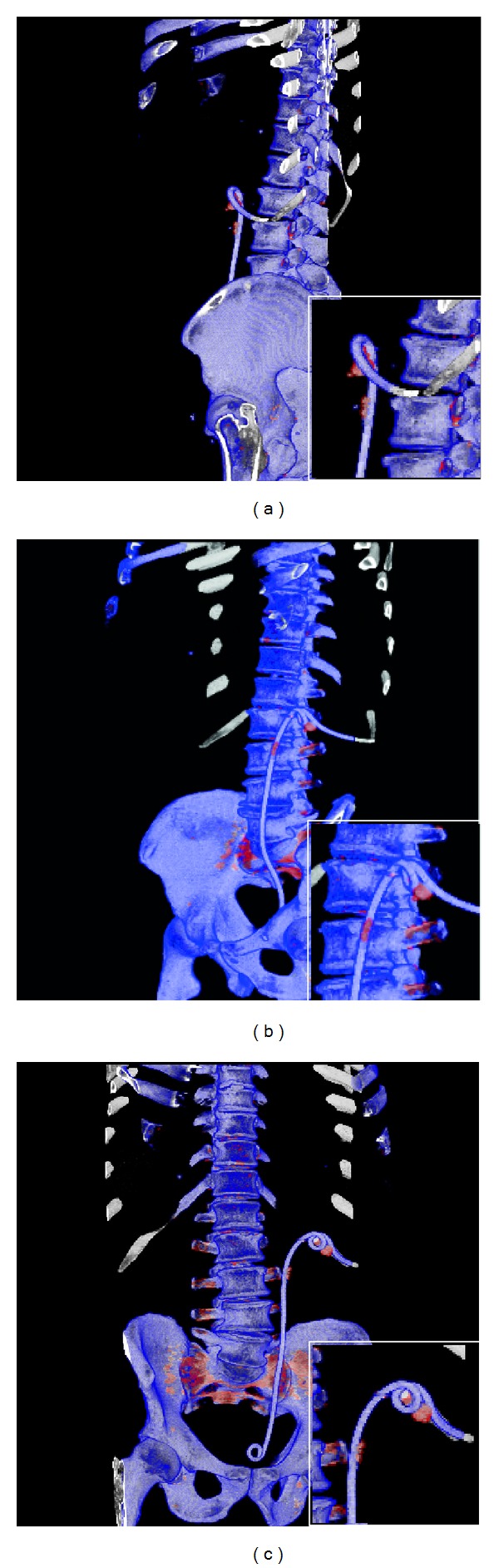
Three-dimensional dual-energy computed tomography (3D DECT) images, viewed from different angles ((a), (b), and (c)) to identify the stone/stent boundary and assess the stone's shape and volume.

Residual 1.1 × 1.3 cm stone fragment and multiple stone fragments adjacent to a nephroureteral stent were noted. Three-dimensional DECT reconstruction improved visualization of the stent/stone boundary by allowing observation from different angles. The stones were characterized by DECT as UA, which led to successful medical treatment of the residual stone and precluded further surgical intervention.  Figure 1 shows the capability of DECT to characterize (and color code) the calculi and stent, compared to concurrent conventional CT images, while Figure 2 demonstrates the enhanced facility afforded by the 3D reconstruction to identify stone burden, shape, and boundary. 

## 3. Discussion

DECT is capable of differentiating renal calculi based on the ratio of X-ray attenuations at different tube potentials, which allows for discrimination between objects that are otherwise indistinguishable when utilizing conventional CT. Moreover, DECT accomplishes such characterization of stone composition without adding to radiation dose [2]. In patients with ureteral stents, DECT also differentiates stent materials, allowing for useful color contrasting between stent and stone [8]. Nevertheless, two-dimensional DECT images may be inadequate when attempting to identify the stone/stent boundary or assess the stone volume. Although three-dimensional rendering of conventional CT images has been shown to provide better organ definition, to our knowledge, the advantages of three-dimensional DECT imaging of renal calculi have not been previously emphasized. 

The present case illustrates the usefulness of three-dimensional DECT for clearly depicting the stone composition, burden, shape, and boundary apart from the adjacent stent. Residual stones and stone fragments following surgical intervention are important predictors of recurrent stone formation, and identifying the stone composition helps determine the best management route (medical treatment versus surgical) [9]. Furthermore, the improved stent/stone visualization with three-dimensional DECT reduces the possibility that a stone or a stone fragment is masked by an adjacent stent that is assigned the same color as the stone, which can lead to the need for follow-up imaging and/or surgical intervention.

## Supplementary Material

The supplementary material shows an animated 3D reconstruction of the DECT images.
